# Higher serum bilirubin levels are associated with hemorrhagic transformation after intravenous thrombolysis in acute ischemic Stroke

**DOI:** 10.3389/fnagi.2023.1159102

**Published:** 2023-04-18

**Authors:** Xiaoqing Chen, Xiangchou Yang, Xia Xu, FangWang Fu, Xiangwei Huang

**Affiliations:** ^1^Department of Clinical Laboratory, The Second Affiliated Hospital and Yuying Children’s Hospital of Wenzhou Medical University, Wenzhou, China; ^2^Department of Hematology and Medical Oncology, The Second Affiliated Hospital and Yuying Children’s Hospital of Wenzhou Medical University, Wenzhou, China; ^3^Department of Neurology, The Second Affiliated Hospital and Yuying Children’s Hospital of Wenzhou Medical University, Wenzhou, China

**Keywords:** bilirubin, hemorrhagic transformation, intravenous thrombolysis, acute ischemic stroke, symptomatic intracranial hemorrhage

## Abstract

**Background:**

Bilirubin has both antioxidative and prooxidative properties. The study aimed to explore the relationship between serum bilirubin and hemorrhagic transformation (HT) after intravenous thrombolysis in patients with acute ischemic stroke.

**Methods:**

The patients receiving intravenous thrombolysis with alteplase were retrospectively analyzed. HT was defined as new intracerebral hemorrhage in follow-up computed tomography images within 24–36 h after thrombolysis. Symptomatic intracranial hemorrhage (sICH) was defined as HT accompanied by worsening neurological function. Multivariate logistic regression and spline regression models were performed to investigate the relationship between serum bilirubin levels and the risk of HT and sICH.

**Results:**

Among 557 included patients, 71 (12.7%) were diagnosed with HT and 28 (5.0%) developed sICH. Patients with HT had significant higher baseline serum total bilirubin, direct bilirubin, and indirect bilirubin levels than those without HT. Multivariable logistic regression analysis indicated that patients with higher serum bilirubin levels, including total bilirubin (OR 1.05, 95% CI 1.01–1.08, *p* = 0.006), direct bilirubin (OR 1.18, 95% CI 1.05–1.31, *p* = 0.004), and indirect bilirubin (OR 1.06, 95% CI 1.02–1.10, *p* = 0.005) had increased risk of HT. Furthermore, multiple-adjusted spline regression models excluded nonlinear association between serum bilirubin levels and HT (*p* > 0.05 for nonlinearity). Similar results were present between serum bilirubin and sICH.

**Conclusion:**

The data showed the positively linearly relationship between serum bilirubin levels and the risk of HT and sICH in patients with acute ischemic stroke undergoing intravenous thrombolysis.

## Introduction

Intravenous thrombolysis with alteplase is the recommended therapy for acute ischemic stroke (AIS) increasing disability-free survival of approximately 5–10% for patients (Emberson et al., 2014). Hemorrhagic transformation (HT) is a common complication after intravenous thrombolysis with a reported prevalence rate of up to 27–37% (Honig et al., 2022). Symptomatic intracerebral hemorrhage (sICH) refers to HT accompanied by neurological deterioration and usually increases the risk of disability and death (He et al., 2022). Considering this, finding biomarkers associated with HT is necessary to help us understand the pathogenesis of HT.

Bilirubin, including direct bilirubin and indirect bilirubin, is the end product of heme catabolism and a potent endogenous antioxidant (Sedlak et al., 2009). A meta-analysis involving 131,450 subjects suggests that higher serum total bilirubin levels are associated with lower risk of ischemic stroke (Zhong et al., 2019). A cross-sectional study showed that patients with prior stroke with higher serum total bilirubin levels less tended to suffer an adverse stroke outcome (Perlstein et al., 2008). The evidence from cohort study showed increased odds of favorable functional outcomes in patients with mild stroke with elevated levels of total bilirubin and direct bilirubin (Duan et al., 2022). However, bilirubin also has neurotoxic effect and numerous studies find that higher serum bilirubin levels are associated with poor functional outcomes in patients with AIS (Wang et al., 2020).

At present, the research on bilirubin and HT after intravenous thrombolysis is scarce. Therefore, we conducted this study to investigate the association of serum bilirubin with HT and sICH after intravenous thrombolysis in patients with AIS. Given the dual antioxidant and prooxidant roles of bilirubin, we further explored whether nonlinear relationship existed between serum bilirubin and HT or sICH.

## Methods

### Patient selection

This was a single-center, retrospective and cohort study. The patients with AIS admitted to the Second Affiliated Hospital of Wenzhou Medical University between January 2017 and January 2022 were examined. We enrolled patients who received intravenous thrombolysis using alteplase (0.9 mg/kg, maximum 90 mg) within 4.5 h after stroke onset. The diagnosis of AIS was confirmed by brain magnetic resonance imaging (MRI) or computed tomography (CT). Patients who had (1) absence of pre-thrombolytic serum bilirubin levels, (2) absence of follow-up CT within 36 h, (3) thrombolysis with low-dose alteplase or interruption of thrombolysis for reasons other than HT, and (4) hepatitis, cirrhosis, liver cancer, biliary obstruction, renal insufficiency, and hemolysis were excluded. The Ethics Committee of the Second Affiliated Hospital of Wenzhou Medical University approved the study. Informed consent was waived for the retrospective nature of the study.

### Data collection

The demographic data (age and sex), medical history (hypertension, diabetes mellitus, hyperlipidemia, atrial fibrillation, coronary artery disease, previous stroke and ongoing antithrombotic therapy) and clinical information (onset-to-treatment time [OTT], National Institutes of Health Stroke Scale [NIHSS], systolic blood pressure, diastolic blood pressure, endovascular treatment and stroke etiology) were collected. Blood samples were collected from the peripheral vein of each patient before the use of alteplase, including blood glucose, platelet, international normalized ratio (INR), creatinine, hemoglobin A1c (HbA1c), total cholesterol (TC), low-density lipoprotein cholesterol (LDL-C), albumin, uric acid, total bilirubin, direct bilirubin, and indirect bilirubin. The stroke etiology was determined according to the Trial of Org 10,172 in Acute Stroke Treatment (TOAST) classification. Serum bilirubin levels were measured by a solid-phase chemiluminescent immunometric assay on the SEIMENS ADVIA2400 automatic biochemical analyzer (SIEMENS AG FWB, Munich, Germany).

### Outcome assessment

Patients were followed up for HT and sICH within 36 h after intravenous thrombolysis. HT was defined as a new hemorrhage occurred on the follow-up CT that was not detected by initial CT before intravenous thrombolysis. According to the National Institute of Neurological Disorders and Stroke (NINDS) study, sICH was defined as HT accompanied by deterioration of neurological function (National Institute of Neurological and Stroke rt-PA Stroke Study Group, 1995). HT can be classified into hemorrhagic infarction (HI) and parenchymal hemorrhage (PH) based on imaging features on CT (Hacke et al., 1995). HI was defined as petechiae within the infarcted area and PH was a more severe HT subtype and defined as blood clot with space-occupying effect. Images were independently reviewed by two investigators (XC and XY) who were blinded to clinical data. In cases of disagreement, the opinion of a third reviewer (FF) was considered.

### Statistical analysis

The normality of distributions for continuous variables was assessed with Q-Q plot and Kolmogorov–Smirnov test. Continuous variables were presented as mean ± standard deviations (SD) in the case of normally distributed data, or medians (interquartile range [IQR]) in the case of skewed data. The intergroup differences in continuous variables were analyzed with unpaired *t*-test, Mann–Whitney *U*-test or Kruskal-Wallis test, where appropriate. Categorical variables were presented as absolute values (percentages). The intergroup differences in categorical variables were analyzed with Pearson’s chi-square test or Fisher’s exact probabilities test, where appropriate.

Logistic regression model was used to explore the association of serum bilirubin with HT and sICH. Age, sex and other variables with *p* < 0.1 in the univariate analysis were included in the multivariate logistic regression model. We included stroke etiology and excluded atrial fibrillation in the model because atrial fibrillation constituted the majority of cardioembolism. Serum bilirubin levels were collapsed into quartiles, and the first quartile values were used as the reference category for the logistic regression analysis. Different serum bilirubin subtypes, including total bilirubin, direct bilirubin and indirect bilirubin, were entered into the models, respectively. Spline regression models were constructed to test whether there was a nonlinear relationship between serum bilirubin and HT or sICH. We set four knots (5th, 35th, 65th, 95th percentiles) in the models and the reference point was the median of the first quartile of serum bilirubin. The SPSS 25.0 (IBM, Armonk, NY, United States) and R 4.2.1 (R Foundation for Statistical Computing, Vienna, Austria) were used for statistical analyses. A two-sided *p* value <0.05 was considered statistically significant.

## Results

A total of 557 patients with AIS treated with intravenous thrombolysis were finally enrolled and average age was 70 (61–80) years, including 352 (66.5%) men. The average concentrations of serum total bilirubin, direct bilirubin and indirect bilirubin were 16.2 (11.8–20.5), 3.8 (2.7–5.2) and 12.2 (8.8–15.5) μmol/L, respectively. The mean OTT of the patients was 159.6 ± 56.9 min and there were 47 patients (8.9%) with endovascular treatment Table 1. Of all patients, 71 patients (12.7%) developed HT, comprising 45 (8.1%) with HI and 26 (4.7%) with PH, and 28 (5.0%) sICH.

**Table 1:** Clinical characteristics of patients, stratified by the presence of HT.

	All (*n* = 557)	HT (*n* = 71)	Non-HT (*n* = 486)	*p*
*Demographic characteristics*				
Age, years (IQR)	70 (61–80)	76 (62–81)	70 (61–80)	0.052
Male, *n* (%)	352 (66.5%)	42 (59.2%)	325 (66.9%)	0.20
*Medical history*				
Hypertension, *n* (%)	411 (77.7%)	59 (83.1%)	375 (77.2%)	0.26
Diabetes mellitus, *n* (%)	145 (27.4%)	18 (25.4%)	134 (27.6%)	0.70
Hyperlipidemia, *n* (%)	166 (31.4%)	21 (29.6%)	153 (31.5%)	0.75
Atrial fibrillation, *n* (%)	145 (27.4%)	41 (57.7%)	122 (25.1%)	**<0.001**
Coronary artery disease, *n* (%)	46 (8.7%)	7 (9.9%)	40 (8.2%)	0.65
Previous stroke, *n* (%)	70 (13.2%)	10 (14.1%)	63 (13.0%)	0.79
Ongoing antithrombotic therapy, *n* (%)	80 (15.1%)	11 (15.5%)	71 (14.6%)	0.84
*Clinical information*				
OTT, min (mean ± SD)	159.6 ± 56.9	159.6 ± 58.2	159.6 ± 56.7	1.00
NIHSS score (IQR)	7 (4–12)	12 (7–17)	6 (4–11)	**<0.001**
Baseline SBP, mmHg (mean ± SD)	159.0 ± 23.7	158.2 ± 27.7	159.1 ± 23.1	0.78
Baseline DBP, mmHg (mean ± SD)	88.3 ± 16.2	89.7 ± 17.3	88.1 ± 16.1	0.44
Endovascular treatment	47 (8.9%)	11 (15.5%)	42 (8.6%)	0.066
Stroke etiology				**<0.001**
Large artery atherosclerosis	164 (31.0%)	18 (25.4%)	148 (30.5%)	
Cardioembolism	147 (27.8%)	37 (52.1%)	125 (25.7%)	
Small vessel occlusion	133 (25.1%)	4 (5.6%)	131 (27.0%)	
Other determined	9 (1.7%)	3 (4.2%)	8 (1.6%)	
Undetermined	76 (14.4%)	9 (12.7%)	74 (15.2%)	
*Laboratory results*				
Baseline blood glucose, mmol/L (IQR)	7.06 (6.05–9.15)	7.26 (6.25–9.63)	7.02 (6.01–8.96)	0.26
Platelet, 10^9^/L (mean ± SD)	207.2 ± 67.0	195.5 ± 52.9	208.7 ± 68.7	0.12
INR (IQR)	1.02 (0.98–1.08)	1.06 (1.01–1.11)	1.02 (0.97–1.07)	**<0.001**
Creatinine, umol/L (IQR)	72 (61–86)	72 (62–90)	72 (61–85)	0.47
HbA1c, % (IQR)	5.80 (5.45–6.40)	5.80 (5.50–6.29)	5.80 (5.44–6.40)	0.94
TC, mmol/L (mean ± SD)	4.45 ± 1.09	4.33 ± 1.06	4.46 ± 1.09	0.35
LDL_C, mmol/L (mean ± SD)	2.76 ± 0.92	2.67 ± 0.99	2.77 ± 0.92	0.41
Albumin, g/L (mean ± SD)	38.3 ± 3.4	38.2 ± 3.5	38.3 ± 3.4	0.67
Uric acid, μmol/L (IQR)	305 (246–369)	259 (211–338)	309 (256–372)	**0.002**
Total bilirubin, μmol/L (IQR)	16.2 (11.8–20.5)	18.0 (13.5–24.7)	15.9 (11.5–20.0)	**0.003**
Direct bilirubin, μmol/L (IQR)	3.8 (2.7–5.2)	4.8 (3.5–6.2)	3.6 (2.7–5.0)	**<0.001**
Indirect bilirubin, μmol/L (IQR)	12.2 (8.8–15.5)	13.5 (9.9–18.3)	12.0 (8.6–15.3)	**0.015**

DBP, diastolic blood pressure; HbA1c, hemoglobin A1c; HT, hemorrhagic transformation; INR, international normalized ratio; IQR, interquartile range; LDL_C, low-density lipoprotein cholesterol; NIHSS, National Institutes of Health Stroke Scale; OTT, onset-to-treatment time; SBP, systolic blood pressure; SD, standard deviations; TC, total cholesterol. The bold values represent significant results in univariate analysis (*p* < 0.05).

**Figure 1:**
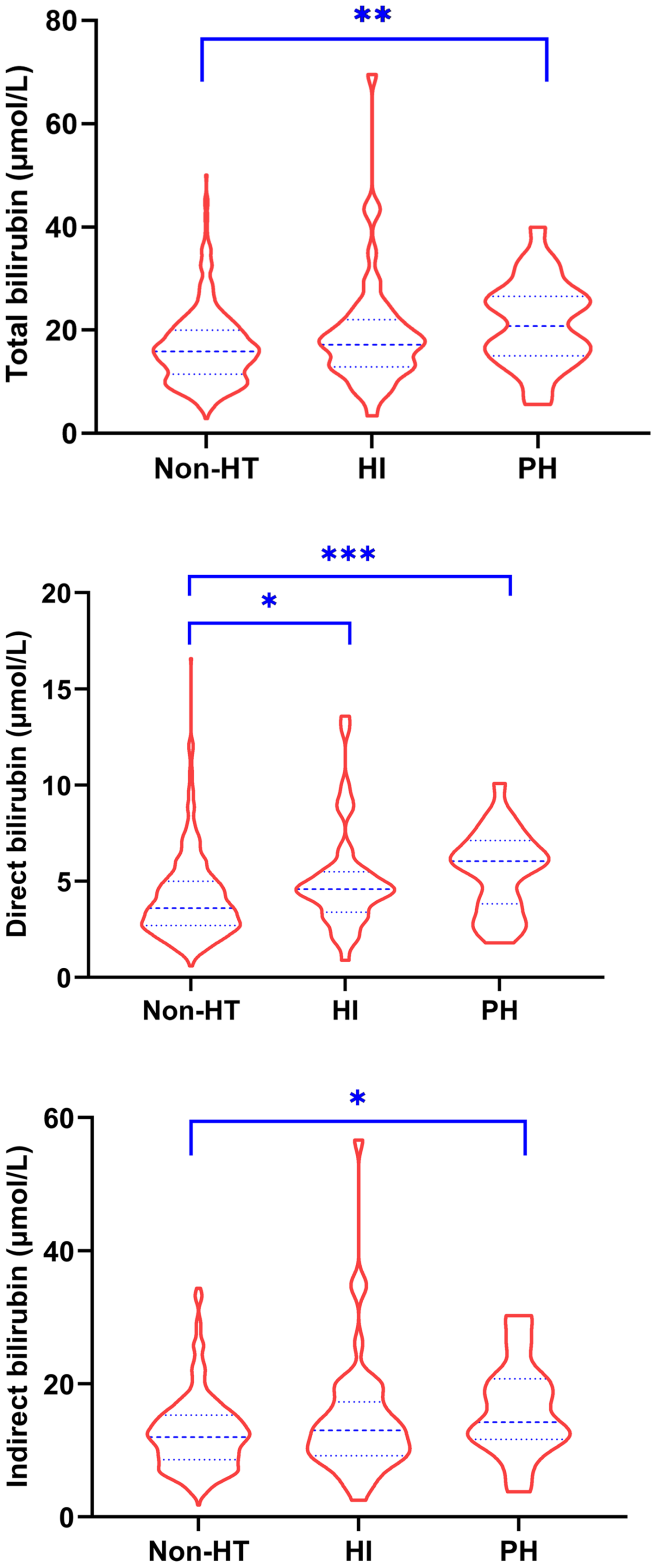
Comparison of serum bilirubin levels in patients without HT and with HI and PH. HI, hemorrhagic infarction; HT, hemorrhagic transformation; PH, parenchymal hemorrhage. * *p* < 0.05, ** *p* < 0.01, *** *p* < 0.001.

The baseline characteristics of patients stratified by the presence of HT and sICH were shown in Tables 1, 2, respectively. The levels of total bilirubin (*p* = 0.003), direct bilirubin (*p* < 0.001) and indirect bilirubin (*p* = 0.015) in patients with HT were significantly higher than those without HT. Similarly, patients with sICH had significantly higher levels of total bilirubin (*p* = 0.013), direct bilirubin (*p* = 0.001) and indirect bilirubin (*p* = 0.026) than patients without sICH. Compared to patients without HT, those with HI had significantly higher levels of direct bilirubin (*p* = 0.017) and those with PH had significantly higher levels of total bilirubin (*p* = 0.008), direct bilirubin (*p* = 0.001) and indirect bilirubin (*p* = 0.020) (Figure 1). In addition, a significantly higher proportion of atrial fibrillation and higher NIHSS scores, INRs and uric acid levels were observed in patients with HT compared to those without HT, while a significantly higher proportion of atrial fibrillation and endovascular treatment and higher INRs and uric acid levels were observed in patients with sICH compared to those without sICH.

**Table 2:** Clinical characteristics of patients, stratified by the presence of sICH.

	sICH (*n* = 28)	Non-sICH (*n* = 529)	*p*
*Demographic characteristics*			
Age, years (IQR)	76 (59–80)	70 (61–80)	0.55
Male, *n* (%)	15 (53.6%)	352 (66.5%)	0.16
*Medical history*			
Hypertension, *n* (%)	23 (82.1%)	411 (77.7%)	0.58
Diabetes mellitus, *n* (%)	7 (25.0%)	145 (27.4%)	0.78
Hyperlipidemia, *n* (%)	8 (28.6%)	166 (31.4%)	0.76
Atrial fibrillation, *n* (%)	18 (64.3%)	145 (27.4%)	**<0.001**
Coronary artery disease, *n* (%)	1 (3.6%)	46 (8.7%)	0.55
Previous stroke, *n* (%)	3 (10.7%)	70 (13.2%)	0.70
Ongoing antithrombotic therapy, *n* (%)	2 (7.1%)	80 (15.1%)	0.25
*Clinical information*			
OTT, min (mean ± SD)	151.5 ± 59.9	160.1 ± 56.7	0.44
NIHSS score (IQR)	11 (5–15)	7 (4–12)	0.092
Baseline SBP, mmHg (mean ± SD)	159.9 ± 29.8	159.0 ± 23.4	0.84
Baseline DBP, mmHg (mean ± SD)	92.1 ± 15.5	88.1 ± 16.2	0.20
Endovascular treatment	6 (21.4%)	47 (8.9%)	**0.027**
Stroke etiology			**0.005**
Large artery atherosclerosis	2 (7.1%)	164 (31.0%)	
Cardioembolism	15 (53.6%)	147 (27.8%)	
Small vessel occlusion	2 (7.1%)	133 (25.1%)	
Other determined	2 (7.1%)	9 (1.7%)	
Undetermined	7 (25.0%)	76 (14.4%)	
*Laboratory results*			
Baseline blood glucose, mmol/L (IQR)	7.29 (6.19–9.34)	7.05 (6.04–9.06)	0.72
Platelet, 10^9^/L (mean ± SD)	185.2 ± 55.7	208.2 ± 67.4	0.077
INR (IQR)	1.08 (1.00–1.14)	1.02 (0.97–1.07)	**0.007**
Creatinine, umol/L (IQR)	69 (59–87)	72 (61–86)	0.46
HbA1c, % (IQR)	5.70 (5.52–6.22)	5.80 (5.45–6.40)	0.65
TC, mmol/L (mean ± SD)	4.14 ± 1.05	4.46 ± 1.09	0.14
LDL_C, mmol/L (mean ± SD)	2.64 ± 0.98	2.77 ± 0.92	0.51
Albumin, g/L (mean ± SD)	38.2 ± 3.9	38.3 ± 3.4	0.87
Uric acid, μmol/L (IQR)	237 (180–336)	309 (252–370)	**0.004**
Total bilirubin, μmol/L (IQR)	18.7 (14.2–28.1)	16.1 (11.8–20.3)	**0.013**
Direct bilirubin, μmol/L (IQR)	5.5 (3.6–7.2)	3.7 (2.7–5.1)	**0.001**
Indirect bilirubin, μmol/L (IQR)	14.2 (10.7–20.5)	12.0 (8.7–15.4)	**0.026**

DBP, diastolic blood pressure; HbA1c, hemoglobin A1c; INR, international normalized ratio; IQR, interquartile range; LDL_C, low-density lipoprotein cholesterol; NIHSS, National Institutes of Health Stroke Scale; OTT, onset-to-treatment time; SBP, systolic blood pressure; SD, standard deviations; sICH, symptomatic intracranial hemorrhage; TC, total cholesterol. The bold values represent significant results in univariate analysis (*p* < 0.05).

**Figure 2:**
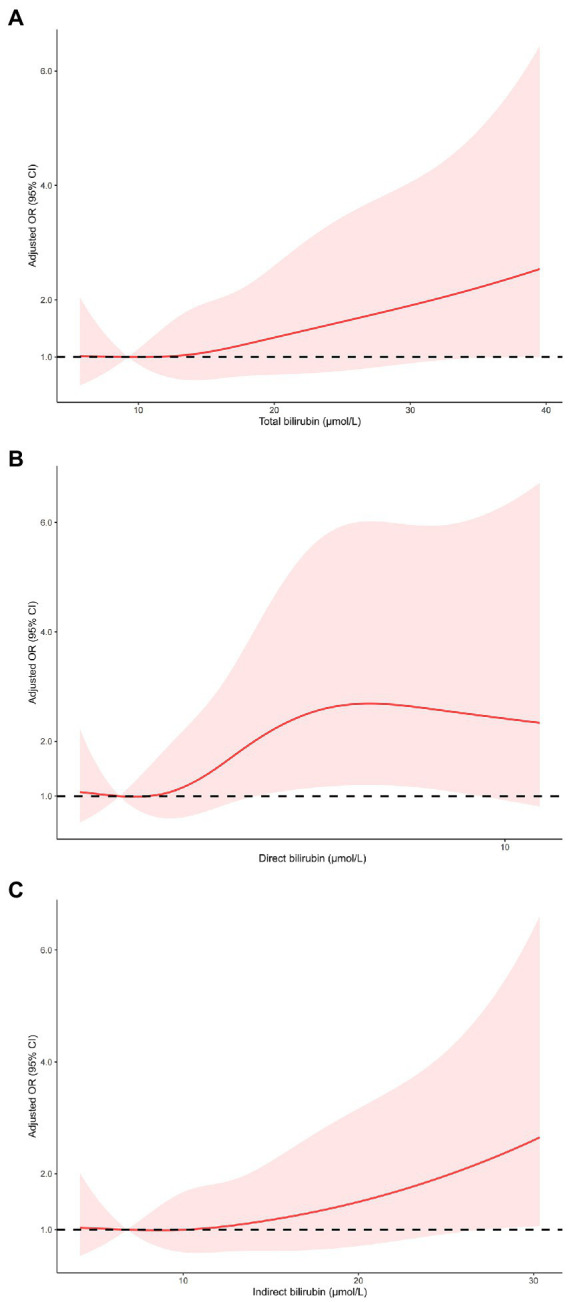
Restricted cubic spline regression models of the relationship between serum bilirubin levels and the risk of HT. Total bilirubin (**A**), direct bilirubin (**B**) and indirect bilirubin (**C**). The model was adjusted for the same confounding variables as in multivariable logistic regression model. HT, hemorrhagic transformation.

**Figure 3:**
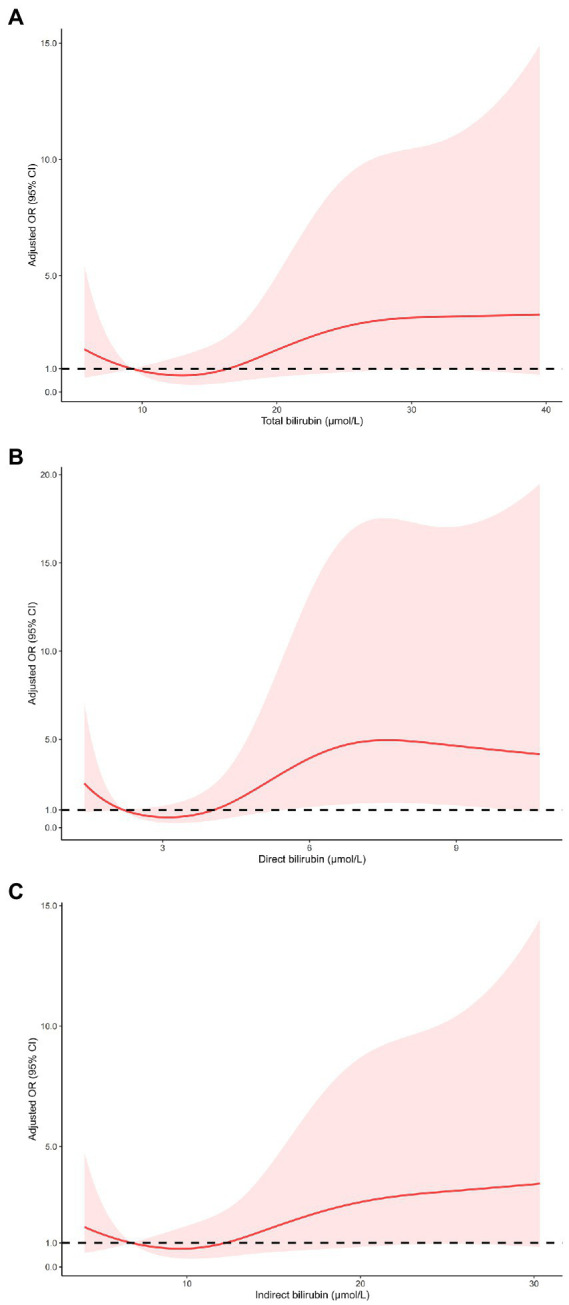
Restricted cubic spline regression models of the relationship between serum bilirubin levels and the risk of sICH. Total bilirubin (**A**), direct bilirubin (**B**) and indirect bilirubin (**C**). The model was adjusted for the same confounding variables as in multivariable logistic regression model. sICH, symptomatic intracranial hemorrhage.

As shown in Table 3, multivariable logistic regression analysis showed that serum bilirubin was independently associated with HT and sICH. For the effect of bilirubin on HT, compared with the first quartile of total bilirubin, the fully adjusted ORs from the second to the fourth quartile were 2.14 (0.88–5.19), 2.51 (1.05–6.00), and 3.36 (1.46–7.72), respectively (*p* for trend = 0.004). The fully adjusted ORs from the second to the fourth quartile of direct bilirubin were 1.34 (0.51–3.50), 2.79 (1.16–6.72), and 4.18 (1.74–10.00), respectively (*p* for trend <0.001), compared with the first quartile. As for indirect bilirubin, the fully adjusted ORs from the second to the fourth quartile were 1.54 (0.70–3.37), 1.13 (0.49–2.56), and 2.37 (1.12–5.03), respectively (*p* for trend = 0.047), compared with the first quartile. The risk of sICH only increased in the fourth quartile of total bilirubin (OR 4.99 95% CI 1.32–18.94), direct bilirubin (OR 7.13, 95% CI 1.84–27.73) and indirect bilirubin (OR 3.33, 95% CI 1.08–10.25) compared with the first quartile of counterparts. Restricted cubic spline regression model excluded the nonlinear association of serum bilirubin with HT and sICH (*p* for nonlinearity >0.05, Figures 2, 3).

**Table 3:** Logistic regression analysis to identify associations of serum bilirubin with HT and sICH.

	HT				sICH			
	Crude model	*p*	Adjusted model	*p*	Crude model	*p*	Adjusted model	*p*
Total bilirubin								
Q1 (≤11.8 μmol/L)	Reference		Reference		Reference		Reference	
Q2 (11.9–16.2 μmol/L)	1.83 (0.78–4.30)	0.16	2.14 (0.88–5.19)	0.092	2.00 (0.49–8.16)	0.33	1.96 (0.46–8.42)	0.36
Q3 (16.3–20.5 μmol/L)	2.36 (1.03–5.43)	0.043	2.51 (1.05–6.00)	0.038	2.48 (0.63–9.79)	0.20	3.01 (0.73–12.43)	0.13
Q4 (≥20.6 μmol/L)	3.54 (1.60–7.85)	0.002	3.36 (1.46–7.72)	0.004	4.35 (1.20–15.77)	0.025	4.99 (1.32–18.94)	0.018
Per 1-μmol/L increase	1.05 (1.02–1.08)	0.001	1.05 (1.01–1.08)	0.006	1.05 (1.01–1.09)	0.014	1.07 (1.02–1.13)	0.010
Direct bilirubin								
Q1 (≤2.7 μmol/L)	Reference		Reference		Reference		Reference	
Q2 (2.8–3.8 μmol/L)	1.48 (0.58–3.80)	0.41	1.34 (0.51–3.50)	0.55	1.41 (0.31–6.43)	0.66	1.12 (0.23–5.38)	0.89
Q3 (3.9–5.2 μmol/L)	3.18 (1.36–7.44)	0.008	2.79 (1.16–6.72)	0.022	1.83 (0.43–7.82)	0.41	1.71 (0.38–7.66)	0.42
Q4 (≥5.3 μmol/L)	5.05 (2.23–11.43)	<0.001	4.18 (1.74–10.00)	0.001	6.30 (1.79–22.15)	0.004	7.13 (1.84–27.73)	0.005
Per 1-μmol/L increase	1.21 (1.10–1.34)	<0.001	1.18 (1.05–1.31)	0.004	1.24 (1.08–1.41)	0.002	1.29 (1.10–1.50)	0.002
Indirect bilirubin								
Q1 (≤8.8 μmol/L)	Reference		Reference		Reference		Reference	
Q2 (8.9–12.2 μmol/L)	1.46 (0.69–3.12)	0.32	1.54 (0.70–3.37)	0.29	1.23 (0.37–4.12)	0.74	1.26 (0.36–4.43)	0.72
Q3 (12.3–15.5 μmol/L)	1.09 (0.49–2.40)	0.84	1.13 (0.49–2.56)	0.78	1.00 (0.28–3.53)	1.00	1.16 (0.32–4.27)	0.82
Q4 (≥15.6 μmol/L)	2.41 (1.18–4.92)	0.016	2.37 (1.12–5.03)	0.024	4.35 (0.93–7.93)	0.067	3.33 (1.08–10.25)	0.036
Per 1-μmol/L increase	1.06 (1.02–1.10)	0.002	1.06 (1.02–1.10)	0.005	1.06 (1.01–1.11)	0.021	1.07 (1.02–1.13)	0.010

Adjusted Model: adjusted for age, sex, NIHSS score, endovascular treatment, stroke etiology, INR, and uric acid.

HT, hemorrhagic transformation; INR, international normalized ratio; NIHSS, National Institutes of Health Stroke Scale; sICH, symptomatic intracranial hemorrhage.

## Discussion

The main finding of the present study was that elevated levels of serum bilirubin, including total bilirubin, direct bilirubin, and indirect bilirubin, were independently linearly associated with the risk of HT and sICH in patients with AIS undergoing intravenous thrombolysis.

The association of bilirubin and HT in patients with AIS has been reported in the past, but not in those receive intravenous thrombolysis. Tan et al. found that high serum total bilirubin levels were associated with HT in general patients with AIS but the proportion of patients with intravenous thrombolysis in this study was only 1.8% (Tan et al., 2016). Jian et al. (2020) enrolled patients with AIS with mechanical thrombectomy and measured the serum levels of different subtypes of bilirubin. The research findings showed that high levels of total bilirubin, direct bilirubin, and indirect bilirubin were independent risk factors of HT, while high levels of total bilirubin and indirect bilirubin were independent risk factors of sICH. Another study focusing on patients with AIS with intravenous thrombolysis reported the null relationship between any subtypes of bilirubin and sICH defined by NINDS criteria (Peng et al., 2022). The findings in our study are partly consisted with previous work, and larger cohorts are needed to examine the association between bilirubin and sICH.

The potential mechanism that how serum bilirubin influences the risk of HT in patients with AIS receiving intravenous thrombolysis is unclear. Blood–brain barrier (BBB) disruption is a pivotal process in HT resulting in blood extravasation through fragile vessels (Arba et al., 2020). Generation of reactive oxygen species and subsequent oxidative stress during acute stage of ischemic stroke damage BBB integrity (Abdullahi et al., 2018). Although bilirubin has beneficial antioxidant effects, such potent antioxidant sometimes behaves in a toxic manner (Soto Conti, 2021). An *in vitro* study demonstrated a time-dependent dual effect of bilirubin on the integrity of human brain microvascular endothelial cells. Early exposure (4 h) to high levels of indirect bilirubin increased number of caveolae and levels of caveolin-1 and vascular endothelial growth factor, while prolonged exposure (72 h) disruption led to down-regulation of zonula occludens-1 and β-catenin and thereby damage the tight junctions and cell-to-cell contacts (Palmela et al., 2012). Oxidative stress plays an important role in neurotoxic effects of bilirubin (Watchko and Tiribelli, 2013). Thus, bilirubin may cause HT by excessive oxidative stress. Although there are dual effects of bilirubin, the spline regression analysis confirmed no nonlinear relationship between serum bilirubin and HT.

As a physiological antioxidant, serum bilirubin levels were increased at acute stage of ischemic stroke in response to oxidative stress. The extent of elevated serum bilirubin levels is positively related to the severity of stroke (Wang et al., 2020). Taking stroke severity (NIHSS score) into account, our study still showed that high serum bilirubin levels were associated with increased risk of HT. A few studies reported that patients with AIS with elevation of serum bilirubin levels had lower risk of early neurological deterioration (Sheng et al., 2021) and increased odds of good functional outcomes (Duan et al., 2022). However, more studies revealed that elevated serum bilirubin levels were independent predictors of poor functional outcome after AIS (Wang et al., 2018; Ouyang et al., 2021; Peng et al., 2022). The findings suggest the predominant role of bilirubin in acute stage of ischemic stroke is a prooxidant, which support our research results.

The significant difference in serum bilirubin levels between HI and non-HT was only observed in direct bilirubin. This implies that direct bilirubin is superior to total bilirubin and indirect bilirubin for predicting HT after intravenous thrombolysis. Direct bilirubin is more soluble in serum than lipophilic indirect bilirubin after conjugation and bound weakly to albumin, thus making direct bilirubin an active form of bilirubin more readily available than indirect bilirubin (Hansen et al., 2020).

There are several limitations to our study. First, this is a retrospective and single-center study with a moderate sample size. The conclusion of our study needs to be verified in larger cohorts. Second, serum bilirubin was measured at admission and serial bilirubin measurements were not obtained. Whether dynamics of serum bilirubin are associated with HT is unknown. Third, different definition of sICH may have impact on the association between bilirubin and sICH. Due to the retrospective nature of this study, we only adopted one definition of sICH. Finally, functional outcomes and cognitive performance were not evaluated in our study. The impact of serum bilirubin on long-term prognosis in patients with AIS needs further investigation.

## Conclusion

In conclusion, higher serum bilirubin (total bilirubin, direct bilirubin, and indirect bilirubin) levels at admission are associated with HT and sICH after intravenous thrombolysis in patients with AIS. Future studies are needed to clarify the pathophysiological mechanism underlying the association of bilirubin with HT.

## Data availability statement

The raw data supporting the conclusions of this article will be made available by the authors, without undue reservation.

## Ethics statement

The studies involving human participants were reviewed and approved by The Ethics Committee of the Second Affiliated Hospital of Wenzhou Medical University. Written informed consent for participation was not required for this study in accordance with the national legislation and the institutional requirements. Written informed consent was not obtained from the individual(s) for the publication of any potentially identifiable images or data included in this article.

## Author contributions

XH and FF conceived and designed the study. All authors acquired the data, which XC and XY analyzed. XY, XX, XH, and FF assisted in data interpretation and XC wrote the manuscript. All authors contributed to the article and approved the submitted version.

## Conflict of interest

The authors declare that the research was conducted in the absence of any commercial or financial relationships that could be construed as a potential conflict of interest.

## Publisher’s note

All claims expressed in this article are solely those of the authors and do not necessarily represent those of their affiliated organizations, or those of the publisher, the editors and the reviewers. Any product that may be evaluated in this article, or claim that may be made by its manufacturer, is not guaranteed or endorsed by the publisher.
